# Physical literacy across years of study: evidence from Slovenian higher education

**DOI:** 10.3389/fspor.2026.1922726

**Published:** 2026-07-30

**Authors:** Črtomir Matejek, David Kukovica, Tina Vršnik Perše

**Affiliations:** Faculty of Education, University of Maribor, Maribor, Slovenia

**Keywords:** cognitive domain, emerging adulthood, health promotion, higher education, physical activity, physical literacy, university students

## Abstract

**Introduction:**

Physical literacy is increasingly conceptualized as a multidimensional and lifelong construct integrating physical competence, motivation, confidence, knowledge, understanding, and behavioral engagement in physical activity. Although the construct has been widely examined among children and adolescents, less is known about differences in physical literacy across years of higher education.

**Methods:**

This cross-sectional study investigated whether year of study is associated with differences in physical literacy domains among Slovenian university students. A total of 302 students enrolled in the first to fourth years of study at Slovenian higher education institutions completed the Slovenian 41-item adaptation of the College Student Physical Literacy Questionnaire. One-way ANOVA and Scheffe post-hoc tests were used to examine differences across study years.

**Results:**

The largest differences were observed in the Cognitive Domain, followed by cognition of physical activity and cognition of healthy lifestyle. Significant differences were also found for physical activity, motivation to participate in physical activity, and the Behavioral Domain. In contrast, no significant differences were observed for the Physical Domain, Emotional Domain, sedentary behavior, or confidence. Post-hoc analyses indicated that the most consistent differences occurred between first- and second-year students.

**Discussion:**

The findings suggest that differences across years of university study are primarily evident in cognitive and behavioral components of physical literacy, whereas perceived physical competence and emotional components remain comparatively stable. Universities may therefore represent important settings for supporting lifelong physical literacy through curricular and extracurricular strategies that strengthen knowledge, awareness, motivation, and active behavioral engagement.

## 1 Introduction

Physical literacy has become a central concept in contemporary physical education, public health, sport pedagogy, and lifelong physical activity research. Rather than describing a narrow set of motor skills or fitness outcomes, physical literacy refers to the integrated development of motivation, confidence, physical competence, knowledge, and understanding that enables individuals to value and take responsibility for engagement in physical activity throughout life. This multidimensional interpretation is consistent with current attempts to position physical literacy as a lifespan construct, a pedagogical orientation, and a public health resource rather than a single measurable trait.

Several recent theoretical contributions have strengthened this broader view. Rudd et al. (1) framed physical literacy as a journey of individual enrichment shaped through ecological interactions between the person, the task, and the environment. Carl et al. (2) further emphasized the biographical nature of this journey, proposing that physical literacy should be understood through life-course experiences rather than isolated assessments. Complementary review work by Edwards et al. (3), Ryom et al. (4), and Carl et al. (5) shows that measurement approaches have expanded substantially, yet the field still contains conceptual and methodological blank spots, especially among adults and emerging adults.

Higher education represents a particularly important setting for the study of physical literacy. University students are usually in emerging adulthood, a developmental period characterized by increasing autonomy, changing social networks, academic pressures, and reorganization of daily routines. These transitions can alter participation in physical activity, sedentary behavior, health-related knowledge, motivation, and self-perception. The transition into university has been identified as a period in which physical activity may decline, making it a relevant window for physical literacy-oriented interventions. Kwan et al. (6, 7) showed that physical literacy-based programs targeting first-year university students can address the decline in physical activity during the university transition, while Kim and Cardinal (8) demonstrated that policy conditions in instructional physical activity programs are related to psychological and behavioral profiles among freshmen.

A substantial body of university-based research indicates that physical literacy is responsive to educational environments. Choi et al. (9) found that sport education in a required university physical education program improved perceived physical literacy, motivation, and physical activity levels. In a related pedagogical study, Choi et al. (10) argued that sport education can operationalize physical literacy within university physical education by creating meaningful, socially engaging, and student-centered learning experiences. Choi et al. (11) also demonstrated that online and hybrid teaching formats during pandemic-related quarantine affected relationships among physical literacy attributes, highlighting the importance of instructional context. These studies collectively suggest that university education may be associated with differences in physical literacy; however, these differences may vary across different domains.

The cognitive dimension is of particular interest in higher education. University contexts are designed to develop knowledge, understanding, critical thinking, and self-regulation, all of which align closely with cognitive components of physical literacy. Li and Shen (12) linked physical education and sport to intellectual development among college students, while Carl, Sudeck, and Pfeifer (13) conceptualized physical activity-related health competence as a set of competencies needed for a healthy, physically active lifestyle. Jiang et al. (14) further demonstrated relationships among eHealth literacy, physical literacy, and physical activity in Chinese university students, suggesting that health-related knowledge, digital health competence, and physical literacy are interconnected. Such findings support the expectation that differences across years of study may be particularly associated with cognitive physical literacy.

Behavioral dimensions are also central to the construct. Physical literacy is not merely knowledge about movement but also the capacity and disposition to engage in physical activity. Ma et al. (15) reported associations between physical literacy and physical activity among Chinese undergraduates, and Yan et al. (16) examined relationships between physical literacy, physical activity, and sedentary behavior. Zhang et al. (17), in Frontiers in Psychology, found that physical fitness, physical activity, and physical literacy are interrelated among Chinese university students. Research on strategies to enhance undergraduate physical activity levels (18) further underscores that university settings can either support or constrain active behavior. These studies suggest that physical literacy should be investigated through both cognitive and behavioral lenses.

Motivation and affective dispositions are equally important. Ahmed and Al Salim (19) showed that quality physical education can enhance the motive for physical activity and its underlying behavior among university students. Santos et al. (20), in Frontiers in Sports and Active Living, aligned physical literacy with critical positive youth development and student-centered pedagogy, emphasizing autonomy, competence, and meaningful participation. Lynch and Sargent (21) similarly used meaningful physical education as a lens for understanding democratic pedagogy in higher education. At the same time, Van Wyk et al. (22) found that social and emotional dimensions of physical literacy remain underdeveloped in post-secondary curricula, indicating that university programs may still prioritize physical and cognitive aspects over social and emotional development.

Recent studies further support the relevance of physical literacy in educational and lifespan contexts. Fu et al. (23) developed a structural model of university faculty perceived physical literacy and identified evidence-based quality physical education as a key factor in university contexts. Vieira et al. (24) reviewed qualitative evidence on physical literacy in school contexts and emphasized the role of learning environments, meaning, and contextualized experiences. Čurović et al. (25) explored the gap between physical literacy terminology and practical application, suggesting that physical literacy must be translated into concrete pedagogical and institutional practices. Fraile-Martínez et al. (26), in Frontiers in Education, also argued for the integration of physical activity into academic settings, providing broader educational justification for embedding movement, health, and physical activity within formal learning environments.

Curricular and institutional perspectives reinforce these arguments. Yang and Miao (27) and Haojun et al. (28), all emphasized that university physical education should be reformed to support broader competencies, lifelong healthy participation, and holistic student development. Lobo et al. (29) argued that physical culture in higher education can expand individual interest and engagement in lifelong participation. Similarly, Gleddie and Morgan (30) proposed physical literacy praxis as a transformative framework for physical education, while Agans et al. (31) presented the 4D4D4All framework as a holistic model integrating multiple domains of development for all learners. These contributions position physical literacy as both an educational outcome and an organizing principle for contemporary physical education.

Despite the expansion of research on physical literacy, several gaps remain. First, much of the literature still focuses on children and adolescents, while university students and other emerging adult populations remain less frequently examined. Second, several studies analyze physical literacy as a global construct, even though theoretical models emphasize its multidimensional structure. Third, few studies compare students across different years of higher education to determine whether physical literacy domains develop uniformly or selectively. Fourth, European evidence remains limited compared with the rapidly growing body of research from Asian university contexts. Addressing these gaps is important because universities can design targeted interventions only if they understand which physical literacy domains have the highest associations with the year of study.

Therefore, the present study investigates whether year of study is associated with differences in physical literacy domains among Slovenian university students. Particular attention is given to cognitive, behavioral, physical, and emotional components of physical literacy. Based on the literature, it was expected that cognitive and behavioral dimensions would show stronger differences across years of study than physical and emotional dimensions.

## 2 Aim of the study

The primary aim of this study was to investigate whether year of study is associated with differences in physical literacy domains, with particular emphasis on cognitive, behavioral, physical, and emotional components of physical literacy. A secondary aim was to identify specific dimensions of physical literacy that demonstrate the greatest variability across years of university study.

Because the study included both confirmatory and exploratory objectives, not all research questions were accompanied by formal hypotheses.

## 3 Research questions

RQ1. Is year of study associated with differences in physical literacy among university students?.

RQ2. Do physical, behavioral, cognitive, and emotional domains of physical literacy differ according to year of study?.

RQ3. Which physical literacy dimensions demonstrate the strongest variation across years of study? (exploratory research question).

RQ4. Are all domains of physical literacy similarly associated with year of study?.

## 4 Research hypotheses

H1. Physical literacy differs significantly across years of university study.

H2a. Cognitive dimensions of physical literacy will differ across years of university study, with higher-year students expected to report higher scores than first-year students.

H2b. Behavioral dimensions of physical literacy differ significantly across years of study.

H2c. Physical dimensions of physical literacy differ significantly across years of study.

H2d. Emotional dimensions of physical literacy differ significantly across years of study.

H3. Students enrolled in higher years of study are expected to report higher knowledge and understanding related to healthy lifestyles and physical activity than first-year students.

## 5 Materials and methods

### 5.1 Study design

A cross-sectional quantitative research design was employed. Data were collected between November 2025 and March 2026 using an online survey. The study examined whether physical literacy domains and dimensions differed among first-, second-, third-, and fourth-year students.

### 5.2 Participants

A total of 639 individuals accessed the survey invitation, corresponding to all units in the sampling database. Of these, 343 respondents provided sufficient data for eligibility assessment, including 302 who completed the questionnaire and 41 who submitted partially completed surveys. A further 296 records were excluded because respondents either only opened the invitation (*n* = 274) or clicked on the survey link without providing usable responses (*n* = 22). Only fully completed questionnaires (*n* = 302) were included in the final analyses.

The sample included 302 university students recruited from different Slovenian higher education institutions, including members of the University of Maribor, University of Ljubljana, and University of Primorska. Convenience sampling was used. The sample consisted of 48 male students (15.9%) and 254 female students (84.1%).

Eligible participants were undergraduate students enrolled in Slovenian higher education institutions during the study period. Participation was voluntary and based on self-selection following distribution of the online survey. No additional inclusion or exclusion criteria were applied.

Because the primary objective was to compare physical literacy across years of study, potential participant characteristics such as study discipline, previous sport participation, and curricular exposure were not included as covariates in the analyses and should therefore be considered when interpreting the findings.

### 5.3 Instrument

Physical literacy was assessed using the Slovenian 41-item adaptation of the College Student Physical Literacy Questionnaire. The scale included four domains: Physical Domain, Behavioral Domain, Cognitive Domain, and Emotional Domain. The eight secondary dimensions were motor skills, motor abilities, physical activity, sedentary behavior, cognition of healthy lifestyle, cognition of physical activity, motivation to participate in physical activity, and confidence to participate in physical activity. Responses were recorded on a five-point Likert scale, with higher scores indicating higher levels of self-perceived physical literacy.

The internal consistency of the Slovenian version of the Comprehensive Student Physical Literacy Questionnaire (CSPLQ) was assessed using Cronbach's alpha coefficients. Overall, the questionnaire demonstrated satisfactory psychometric properties across its eight theoretical subscales. Cronbach's alpha coefficients ranged from 0.590 to 0.862. Good internal consistency was observed for the Motor abilities (*α* = 0.862), Physical activity (*α* = 0.836), and Cognition of healthy lifestyle (*α* = 0.815) subscales. Acceptable reliability was demonstrated for Motor skills (*α* = 0.795), Confidence to participate in physical activity (*α* = 0.767), Cognition of physical activity (*α* = 0.741), and Motivation to participate in physical activity (*α* = 0.723). The Sedentary behaviour subscale yielded a lower reliability coefficient (*α* = 0.590), which should be interpreted with caution because the construct comprises only two items, and Cronbach's alpha is known to underestimate internal consistency for very short scales. Overall, these findings support the internal consistency of the Slovenian version of the CSPLQ for assessing physical literacy among university students.

### 5.4 Procedure and ethics

Data were collected using the 1KA online survey platform. Participation was voluntary and anonymous. Participants provided informed consent before completing the questionnaire. The study was approved by the Research Ethics Committee, Faculty of Arts, University of Maribor.

### 5.5 Statistical analysis

IBM SPSS Statistics Version 32 was used. Descriptive statistics were calculated for all domains and dimensions. Homogeneity of variances was assessed using Levene tests. One-way ANOVA was used to examine differences across years of study. Scheffe *post-hoc* tests were conducted for outcomes with significant omnibus effects. Effect sizes were reported as eta squared. Statistical significance was set at *p* < .05.

To examine whether the observed differences across years of study remained after accounting for potential demographic confounders, additional analyses of covariance (ANCOVA) were performed for all physical literacy outcomes. In these analyses, year of study was entered as the fixed factor, whereas sex and year of birth (used as a proxy for age) were included in the model as control variables. Before conducting ANCOVA, assumptions regarding homogeneity of regression slopes, homogeneity of variances, normality of residuals, and multicollinearity among predictors were examined and found to be satisfactory. Adjusted group means (estimated marginal means) were compared using Bonferroni-adjusted pairwise comparisons. Partial eta squared (*η*^2^p) was used as the measure of effect size for ANCOVA models.

## 6 Results

A total of 302 students completed the survey and were included in the analyses. The sample characteristics are shown in Table 1. The distribution across study years was balanced enough for comparative analysis, although the sample was predominantly female.

**Table 1:** Characteristics of the study sample.

Year of study	*n*	%	Mean birth year ± SD	Birth year range
1st year	89	29.5	2,004.65 ± 0.79	2,001–2,005
2nd year	81	26.8	2,003.59 ± 1.10	1,998–2005
3rd year	62	20.5	2002.34 ± 1.14	1998–2003
4th year	70	23.2	2000.97 ± 2.30	1988–2003
Total	302	100.0	-	-

The total sample included 48 male students (15.9%) and 254 female students (84.1%).

Bold rows/values indicate the four main physical literacy domains; the rows beneath each domain represent the corresponding dimensions.

Descriptive statistics for all physical literacy domains and dimensions are presented in Table 2. The highest mean scores were observed for motor skills, cognition of healthy lifestyle, and motivation to participate in physical activity, whereas the lowest mean score was observed for sedentary behavior. Overall, the Cognitive Domain and Physical Domain demonstrated the highest average scores, while the Behavioral Domain had the lowest overall mean.

**Table 2:** Descriptive statistics for physical literacy domains and dimensions.

Outcome	*N*	M	SD	Minimum	Maximum
Physical Domain	**302**	**3**.**89**	**0**.**57**	**1**.**58**	**5**.**00**
Motor Skills	302	4.40	0.54	1.60	5.00
Motor Abilities	302	3.37	0.72	1.56	5.00
Behavioral Domain	**302**	**2**.**99**	**0**.**81**	**1**.**00**	**4**.**75**
Physical Activity	302	3.49	1.17	1.00	5.00
Sedentary Behavior	302	2.48	0.79	1.00	4.50
Cognitive Domain	**302**	**3**.**96**	**0**.**48**	**2**.**75**	**5**.**00**
Cognition of Healthy Lifestyle	302	4.32	0.50	2.50	5.00
Cognition of Physical Activity	302	3.60	0.65	1.80	5.00
Emotional Domain	**302**	**3**.**60**	**0**.**58**	**2**.**09**	**4**.**95**
Motivation to Participate in Physical Activity	302	4.02	0.46	2.45	5.00
Confidence to Participate in Physical Activity	302	3.19	0.89	1.00	5.00

Bold rows/values indicate the four main physical literacy domains; the rows beneath each domain represent the corresponding dimensions.

Descriptive statistics according to year of study are presented in Table 3. Students in the second year generally demonstrated higher mean scores in the Cognitive Domain, Behavioral Domain, physical activity, and cognition-related dimensions compared with first-year students, whereas differences between the third- and fourth-year groups were comparatively smaller.

**Table 3:** Descriptive statistics for physical literacy domains and dimensions separated by year.

Outcome	Year 1 (*n* = 89) M ± SD	Year 2 (*n* = 81) M ± SD	Year 3 (*n* = 62) M ± SD	Year 4 (*n* = 70) M ± SD
Physical Domain	**3.84** ± **0.60**	**3.98** ± **0.53**	**3.85** ± **0.59**	**3.88** ± **0.56**
Motor Skills	4.36 ± 0.56	4.45 ± 0.52	4.39 ± 0.53	4.42 ± 0.53
Motor Abilities	3.32 ± 0.76	3.51 ± 0.71	3.30 ± 0.74	3.34 ± 0.66
Behavioral Domain	**2.81** ± **0.81**	**3.17** ± **0.73**	**2.99** ± **0.83**	**3.00** ± **0.83**
Physical Activity	3.17 ± 1.19	3.72 ± 1.07	3.55 ± 1.22	3.59 ± 1.15
Sedentary Behavior	2.46 ± 0.81	2.62 ± 0.74	2.44 ± 0.87	2.41 ± 0.73
Cognitive Domain	**3.79** ± **0.42**	**4.13** ± **0.45**	**3.92** ± **0.50**	**4.02** ± **0.47**
Cognition of Healthy Lifestyle	4.17 ± 0.45	4.45 ± 0.46	4.30 ± 0.57	4.39 ± 0.50
Cognition of Physical Activity	3.41 ± 0.61	3.82 ± 0.68	3.54 ± 0.61	3.66 ± 0.61
Emotional Domain	**3.52** ± **0.60**	**3.70** ± **0.57**	**3.61** ± **0.55**	**3.59** ± **0.56**
Motivation to Participate in Physical Activity	3.89 ± 0.49	4.11 ± 0.44	4.04 ± 0.45	4.06 ± 0.44
Confidence to Participate in Physical Activity	3.15 ± 0.97	3.29 ± 0.87	3.19 ± 0.87	3.12 ± 0.83

Bold rows/values indicate the four main physical literacy domains; the rows beneath each domain represent the corresponding dimensions.

Levene's tests indicated that the assumption of homogeneity of variances was satisfied for all analyzed outcomes. The results of the one-way ANOVA are presented in Table 4.

**Table 4:** Comparison of physical literacy outcomes across years of study using unadjusted (ANOVA) and adjusted (ANCOVA) models controlling for sex and year of birth.

Outcome	Unadjusted model (ANOVA)			Adjusted model (ANCOVA)		
	F(3, 298)	*p*	*η* ^2^	F(3, 296)	*p*	partial η^2^
Physical Domain	**1**.**06**	.**367**	.011*	**3**.**16**	.**025**	.031*
Motor Skills	0.51	.677	.005	2.45	.064	.024*
Motor Abilities	1.38	.250	.014*	3.01	.031	.030*
Behavioral Domain	**2**.**75**	.**043**	.027*	**4**.**61**	.**004**	.045*
Physical Activity	3.45	.017	.034*	7.14	<.001	.068**
Sedentary Behavior	1.11	.344	.011*	1.00	.393	.010*
Cognitive Domain	**8**.**55**	<**.001**	.079**	**9**.**95**	**<.001**	.092**
Cognition of Healthy Lifestyle	5.10	.002	.049*	6.37	<.001	.061**
Cognition of Physical Activity	6.39	<.001	.060**	7.09	<.001	.067**
Emotional Domain	**1**.**49**	.**219**	.015*	**2**.**45**	.**064**	.024*
Motivation to Participate in Physical Activity	3.84	.010	.037*	6.86	<.001	.065**
Confidence to Participate in Physical Activity	0.57	.632	.006	0.71	.545	.007

The unadjusted model was estimated using one-way ANOVA. The adjusted model was estimated using ANCOVA with sex entered as a fixed factor and year of birth entered as a covariate (proxy for age). Effect sizes are reported as eta squared (η^2^) for ANOVA and partial eta squared (partial η^2^) for ANCOVA. Effect sizes were interpreted using conventional benchmarks for η^2^ (32), which are commonly applied to partial η^2^ (33).

* small effect (η^2^ = .01–.059).

** medium effect (η^2^ = .06–.139).

Bonferroni-adjusted comparisons of estimated marginal means were used for follow-up analyses. Statistical significance was set at *p* < .05.

Bold rows/values indicate the four main physical literacy domains; the rows beneath each domain represent the corresponding dimensions.

The strongest omnibus effect was found for the Cognitive Domain, F(3, 298) = 8.55, *p* < .001, *η*^2^ = .079. Significant effects were also found for cognition of physical activity, F(3, 298) = 6.39, *p* < .001, *η*^2^ = .060; cognition of healthy lifestyle, F(3, 298) = 5.10, *p* = .002, *η*^2^ = .049; motivation to participate in physical activity, F(3, 298) = 3.84, *p* = .010, *η*^2^ = .037; physical activity, F(3, 298) = 3.45, *p* = .017, *η*^2^ = .034; and the Behavioral Domain, F(3, 298) = 2.75, *p* = .043, *η*^2^ = .027. No statistically significant differences were found for sedentary behavior, confidence, the Physical Domain, or the Emotional Domain.

To determine whether the observed differences across years of study remained after accounting for demographic characteristics, additional ANCOVA models controlling for sex and year of birth were performed (Table 4). Overall, adjustment for these covariates resulted in only minor changes in the magnitude of the observed effects and did not substantially alter the overall pattern of findings.

Significant differences between years of study remained evident for several cognitive and behavioral dimensions of physical literacy after adjustment. In contrast, differences in Motor Skills were attenuated and no longer reached statistical significance after controlling for sex and year of birth (*F* = 2.449, *p* = .064, *η*^2^*p* = .024). Year of birth emerged as a significant covariate for Motor Skills (*F* = 7.909, *p* = .005), whereas sex showed no statistically significant association (*p* = .060). Across the remaining outcomes, adjustment for sex and age produced only small changes in effect sizes and statistical significance, indicating that the primary findings were robust after accounting for these demographic variables.

Consequently, the adjusted analyses support the interpretation that the observed differences across years of study cannot be explained solely by differences in participants' sex or age.

**Figure 1:**
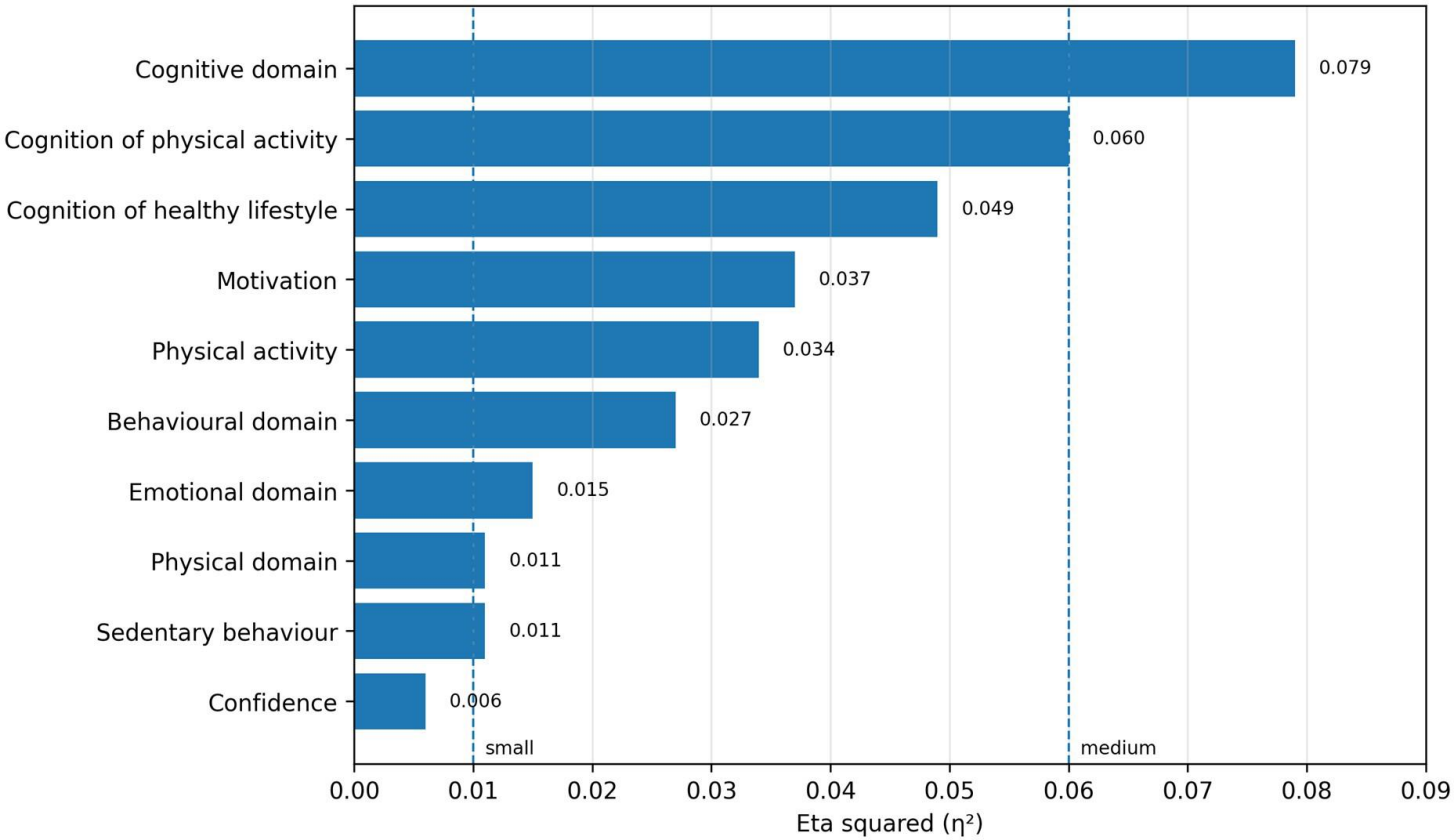
Effect sizes for differences in physical literacy domains and dimensions across years of study. Note. The figure was generated with the assistance of ChatGPT (OpenAI, GPT-5.5) using Python (Matplotlib) for data visualization. The underlying data and visualization specifications were provided and verified by the authors.

The relative magnitude of the observed effect sizes is illustrated in Figure 1, showing that the Cognitive Domain exhibited the largest effect size, followed by Cognition of Physical Activity and Cognition of Healthy Lifestyle.

Scheffe *post-hoc* analyses revealed that second-year students scored significantly higher than first-year students in physical activity (MD = 0.542, *p* = .027), cognition of healthy lifestyle (MD = 0.279, *p* = .004), cognition of physical activity (MD = 0.409, *p* < .001), motivation to participate in physical activity (MD = 0.225, *p* = .017), the Behavioral Domain (MD = 0.352, *p* = .043), and the Cognitive Domain (MD = 0.344, *p* < .001). Additionally, fourth-year students demonstrated significantly higher Cognitive Domain scores than first-year students (MD = 0.230, *p* = .021). No other Scheffe-adjusted pairwise comparisons reached statistical significance.

Taken together, these results indicate that the most consistent differences across years of study occurred in cognitive and behavioral dimensions, particularly between first- and second-year students. The observed pattern indicates that the largest differences across years of study were found between first- and second-year students. These differences may reflect processes occurring during the transition into higher education but cannot be interpreted as evidence of developmental change.

## 7 Discussion

The present study investigated whether physical literacy differed across years of university study among Slovenian university students. Importantly, the present study extends previous university-based research by showing that differences across years of study are not equally distributed across all physical literacy domains. Rather than treating physical literacy as a single global construct, the findings demonstrate a domain-specific pattern, with cognitive outcomes showing the strongest differences. The biggest differences were observed in the cognitive and behavioral dimensions, whereas the physical and emotional domains generally showed weaker associations with year of study.

Additional ANCOVA models demonstrated that the overall pattern of findings remained largely unchanged after accounting for the demographic confounders (sex and year of birth). However, adjustment refined the observed pattern, with several outcomes showing larger partial eta-squared values. Notably, the Physical Domain and Motor Abilities reached statistical significance only after adjustment, suggesting that controlling for demographic covariates reduced unexplained variability and revealed differences that were less apparent in the unadjusted analyses. At the same time, Motor Skills no longer reached statistical significance after adjustment, indicating that this association was partly explained by demographic characteristics. Overall, the adjusted analyses confirmed the general pattern of findings while demonstrating that accounting for demographic variability strengthened several associations and revealed additional statistically significant differences.

The most prominent finding was the significant difference in the Cognitive Domain. This result is consistent with the theoretical premise that higher education is expected to be associated with knowledge, understanding, and informed decision-making. The cognitive pattern observed in the present study aligns with Li and Shen (12), who emphasized the role of physical education and sport in intellectual development, and with Carl et al. (13), who linked physical activity-related health competence to knowledge and self-regulatory competencies. It also corresponds with Jiang et al. (14), who demonstrated relationships among eHealth literacy, physical literacy, and physical activity among university students. These converging findings support the interpretation that cognitive physical literacy appears to differ more consistently across years of study than other domains.

One possible explanation is that university education is inherently knowledge-oriented. Regardless of study discipline, students are continuously exposed to learning environments that promote critical thinking, information processing, health awareness, and self-regulated learning. These educational processes are conceptually aligned with the cognitive dimension of physical literacy and may therefore be reflected in stronger differences across years of study than in domains that depend more heavily on long-term movement experiences or stable personal characteristics.

The significant effects for cognition of physical activity and cognition of healthy lifestyle further strengthen this interpretation. Students in higher years, especially second-year students, reported higher knowledge and understanding related to physical activity principles and healthy lifestyle. These results are consistent with Yang and Miao (27) and Haojun et al. (28), who emphasized that university physical education curricula should support broad health-related competencies rather than only technical sport performance. The present findings extend this literature by showing that such differences are detectable across years of study in a Slovenian higher education context.

The significant differences in physical activity and the Behavioral Domain are also consistent with previous research. Choi et al. (9) showed that sport education improved university students’ perceived physical literacy and physical activity levels, while Choi et al. (10) demonstrated that sport education can operationalize physical literacy in university physical education. Kwan et al. (6, 7) similarly highlighted the importance of physical literacy-based interventions during the transition into university, a period often associated with reductions in physical activity. The present study supports these findings by showing that first-year students had significantly lower physical activity and Behavioral Domain scores than second-year students. This pattern is consistent with differences between first- and second-year students, although the cross-sectional design does not allow conclusions regarding adaptation or individual change over time.

Behavioral differences may similarly reflect changes in students' routines and adaptation to university life. As students become more familiar with the university environment, they may establish more stable daily habits, identify opportunities for physical activity, and develop greater autonomy in managing their lifestyle. Although the present cross-sectional design does not allow conclusions regarding individual change, these mechanisms provide a plausible theoretical explanation for the observed differences across years of study.

Motivation to participate in physical activity also differed significantly between first- and second-year students. This finding aligns with Ahmed and Al Salim (19), who reported that quality physical education can enhance motivation and underlying physical activity behavior. It is also conceptually consistent with Santos et al. (20), who positioned physical literacy within student-centered pedagogy and critical positive youth development. From this perspective, motivation may improve when students encounter more autonomous, meaningful, and supportive movement experiences in university contexts. The present findings suggest that higher motivation was observed among second-year students compared with first-year students in higher education, although the overall Emotional Domain did not differ significantly.

**Figure 2:**
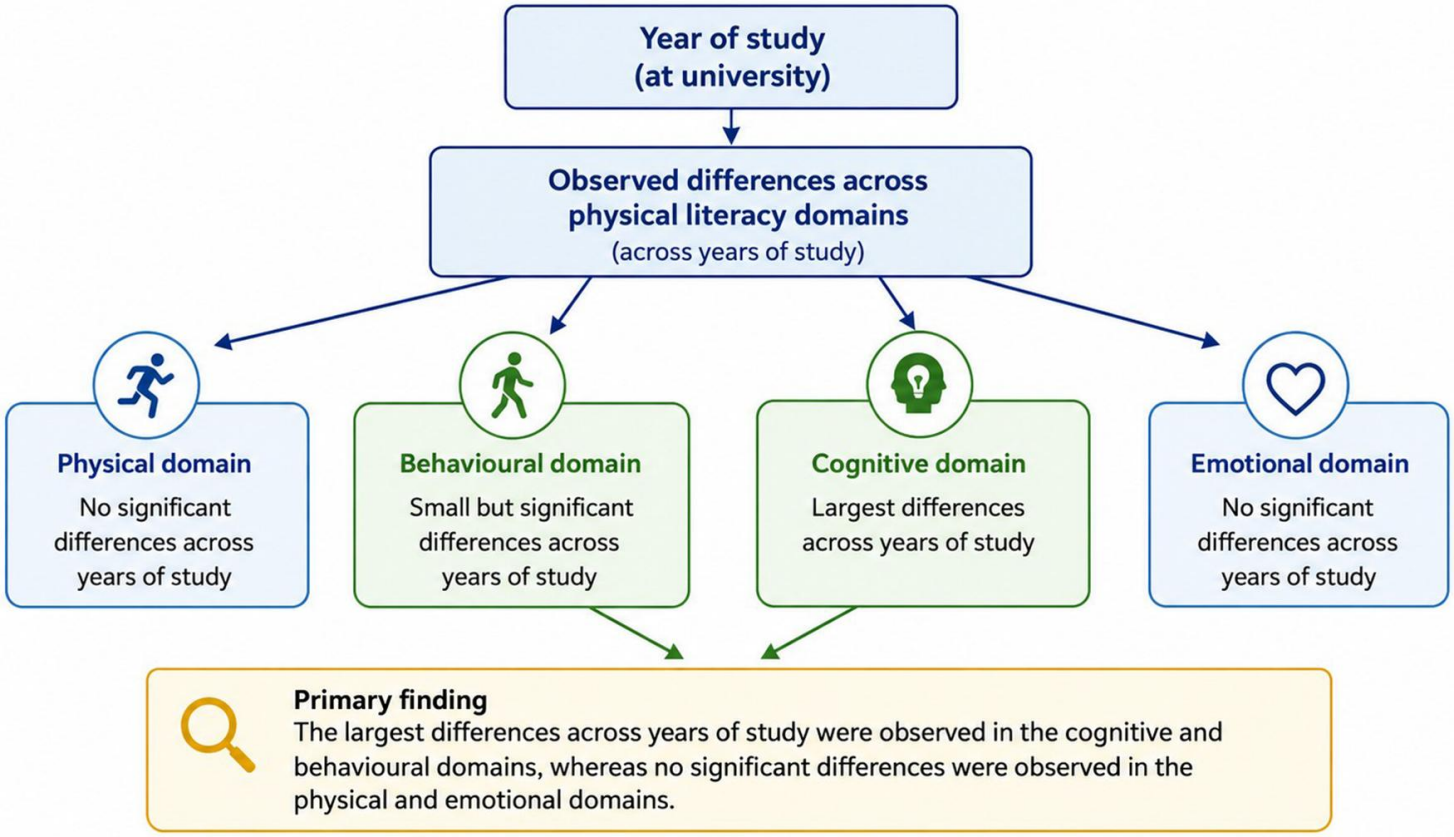
Conceptual model illustrating the observed pattern of associations between year of study and physical literacy domains. Note. The figure was created with the assistance of ChatGPT (OpenAI, GPT-5.5) for conceptual visualization and graphical presentation. The underlying findings, conceptual structure, and content were provided and verified by the authors.

The overall pattern of the findings is summarized in Figure 2, which illustrates that stronger associations with year of study were observed for the cognitive and behavioral domains of physical literacy than for the physical and emotional domains.

The adjusted findings for the Physical Domain and Motor Abilities provide additional insight into the role of demographic covariates. Although these outcomes were not statistically significant in the unadjusted analyses, both reached statistical significance after adjustment, suggesting that controlling for sex and year of birth reduced unexplained variability, thereby improving the detection of differences across years of study. Nevertheless, the adjusted effects remained smaller than those observed for the Cognitive Domain, supporting the conclusion that cognitive aspects of physical literacy appear to be more strongly associated with progression through university education. This interpretation is consistent with life-course perspectives proposed by Rudd et al. (1) and Carl et al. (2), which conceptualize physical literacy as a long-term developmental journey shaped by cumulative experiences. By emerging adulthood, perceived physical competence may be less malleable than knowledge and behavior, especially when university curricula do not systematically target motor competence development. The findings also align with Van Wyk et al. (22), who noted that post-secondary curricula often do not fully integrate all physical literacy domains.

The Emotional Domain and confidence to participate in physical activity did not differ significantly across study years. This finding deserves careful interpretation. On the one hand, motivation showed a significant dimension-level effect; on the other hand, the broader Emotional Domain and confidence remained stable. Emotional aspects of physical literacy, such as confidence and affective engagement, may also be more strongly influenced by long-term personal experiences, social relationships, and previous participation in physical activity than by higher year of study alone. Consequently, these dimensions may show greater stability across years of study than cognitive or behavioral domains. Van Wyk et al. (22) identified social and emotional domains as underrepresented in post-secondary physical literacy curricula, and Čurović et al. (25) emphasized the gap between physical literacy language and practical application. The present findings are consistent with this concern: the findings suggest that knowledge-related outcomes showed stronger associations with year of study than emotional outcomes. Emotional physical literacy may require intentional pedagogical design, inclusive practice, supportive climates, and sustained positive movement experiences.

The findings also contribute to the broader literature on physical literacy in higher education. Fu et al. (23) highlighted the role of evidence-based quality physical education and institutional support in university faculty perceptions of physical literacy. Vieira et al. (24) emphasized that physical literacy is shaped by contextualized learning experiences, while Fraile-Martínez et al. (26) argued for stronger integration of physical activity into academic environments. The present study provides empirical evidence from Slovenian higher education that supports these arguments: higher year of study appears to be associated with cognitive and behavioral development, but not equally with physical and emotional development.

The strongest *post-hoc* pattern occurred between first- and second-year students. This is an important practical finding. The findings suggest that first-year students may represent an appropriate target group for physical literacy promotion. Kwan et al. (6, 7) reached similar conclusions in relation to first-year interventions. Universities may therefore consider implementing physical literacy-oriented programs specifically during the transition into higher education, with emphasis on self-regulation, health knowledge, active routines, and meaningful participation.

Taken together, the findings suggest that different domains of physical literacy may follow distinct patterns across emerging adulthood. Domains closely related to knowledge acquisition and everyday behavioral regulation appear more closely aligned with educational experiences, whereas physical competence and emotional dispositions may reflect longer-term developmental processes extending beyond the university context. This interpretation is consistent with contemporary multidimensional models of physical literacy, which emphasize that its domains may not develop in parallel throughout the life course.

Several limitations should be considered. The cross-sectional design does not allow causal conclusions or inferences about individual developmental trajectories over time. Accordingly, the observed differences across years of study should not be interpreted as evidence of developmental change or the effects of university education on physical literacy, as they may reflect cohort differences rather than within-person change. Longitudinal studies are needed to determine whether these observed differences represent true developmental change or cohort effects. The use of convenience sampling may also have introduced selection bias, as students with a greater interest in physical activity or health-related topics may have been more likely to participate. Consequently, the findings may not fully represent the broader population of Slovenian university students. Furthermore, the predominance of female participants (84.1%) may have influenced the observed associations, as previous research suggests that physical literacy and physical activity patterns may differ by sex. Consequently, the findings should not be assumed to generalize equally to male and female university students. In addition, although the ANCOVA analyses adjusted for sex and year of birth, the analyses did not adjust for several other potentially important confounding variables, such as study discipline, previous sport participation, participation in organized physical activity, socioeconomic background, curricular exposure, or institutional differences. Consequently, the observed associations between year of study and physical literacy outcomes may partly reflect these factors rather than year of study itself. Future studies should employ multivariable analytical approaches and longitudinal designs to better estimate the independent association between year of study and physical literacy outcomes while accounting for these potential confounders. In addition, the study relied on perceived rather than objectively assessed physical literacy. Consequently, the findings reflect students' self-perceptions, which may differ from objectively measured competence or behavior. Future research should combine self-report instruments with objective or performance-based measures and should examine whether curricular exposure, study discipline, prior sport participation, and institutional context moderate associations between year of study and physical literacy outcomes. Finally, because the study was conducted exclusively in Slovenia, the findings may not be directly transferable to higher education systems with different educational structures, cultural contexts, or physical education policies.

Despite these methodological limitations, the study provides one of the few domain-specific examinations of physical literacy across years of university study and therefore offers a useful foundation for future longitudinal investigations. This domain-specific approach is important because it demonstrates that differences in physical literacy across years of study are not uniform across all domains. Instead, cognitive and behavioral components showed stronger associations with year of study than physical and emotional components. This finding has direct implications for higher education institutions seeking to promote lifelong physical activity and health.

## 8 Conclusion

This study provides evidence of associations between year of study and cognitive and behavioral components of physical literacy among Slovenian university students. The largest effect was observed for the Cognitive Domain, with additional significant differences in cognition of physical activity, cognition of healthy lifestyle, physical activity, motivation, and the Behavioral Domain. Physical and emotional domains remained comparatively stable across years of study. The findings also support the view that different domains of physical literacy may respond differently to educational experiences, reinforcing the importance of considering physical literacy as a multidimensional construct.

The *post-hoc* pattern showed that the most consistent differences occurred between first- and second-year students, suggesting that the transition into higher education may be a critical period for physical literacy development. These findings are consistent with the possibility that the transition into higher education is an important period; however, this interpretation should be confirmed in longitudinal studies.

From a practical perspective, universities should not assume that students at different years of study demonstrate similar physical literacy profiles. Instead, higher education institutions should intentionally design curricular and extracurricular strategies that strengthen cognitive understanding, active behavior, motivation, and confidence. Particular attention should be given to first-year students and to the social and emotional domains of physical literacy, which appear less responsive to current educational experiences. Integrating physical literacy principles into university curricula may contribute to healthier, more active, and more physically literate graduates.

Although the present findings may help inform future educational practice, recommendations for curriculum development and institutional interventions should be confirmed through longitudinal and intervention-based research before widespread implementation.

## 9 Practical implications for higher education

- The findings suggest that first-year students may represent an appropriate target group for physical literacy promotion, as the largest differences across years of study were observed between first- and second-year students. However, this possibility should be confirmed in longitudinal studies.
- University physical education may benefit from explicitly address health-related knowledge, physical activity principles, and self-regulation strategies, as cognitive components seem particularly responsive to year of study.
- Universities may consider supporting active behaviors through accessible opportunities for physical activity, active campus initiatives, and integration of movement into academic routines.
- The present findings suggest that greater attention to social and emotional domains may be warranted; however, the effectiveness of specific curricular approaches should be evaluated in future intervention studies.

These practical implications should be interpreted as preliminary considerations derived from cross-sectional associations rather than evidence of intervention effectiveness. Future longitudinal and intervention-based studies are needed to determine whether the proposed educational and institutional strategies lead to improvements in physical literacy.

## Data Availability

The raw data supporting the conclusions of this article will be made available by the authors, without undue reservation.
